# Dose-dependent reduction of somatic expansions but not Htt aggregates by di-valent siRNA-mediated silencing of MSH3 in HdhQ111 mice

**DOI:** 10.1038/s41598-024-52667-3

**Published:** 2024-01-24

**Authors:** Rachelle Driscoll, Lucas Hampton, Neeta A. Abraham, J. Douglas Larigan, Nadine F. Joseph, Juan C. Hernandez-Vega, Sarah Geisler, Fu-Chia Yang, Matthew Deninger, David T. Tran, Natasha Khatri, Bruno M. D. C. Godinho, Garth A. Kinberger, Daniel R. Montagna, Warren D. Hirst, Catherine L. Guardado, Kelly E. Glajch, H. Moore Arnold, Corrie L. Gallant-Behm, Andreas Weihofen

**Affiliations:** 1 Translational Sciences, Biogen, 225 Binney Street, Cambridge, MA 02142 USA; 2 Neurodegenerative Diseases Research Unit, Biogen, 225 Binney Street, Cambridge, MA 02142 USA; 3 Biotherapeutics and Medicinal Sciences, Biogen, 225 Binney Street, Cambridge, MA 02142 USA; 4 Atalanta Therapeutics, 51 Sleeper Street, Boston, MA 02210 USA

**Keywords:** Huntington's disease, Pharmacodynamics

## Abstract

Huntington's disease (HD) is a progressive neurodegenerative disorder caused by CAG trinucleotide repeat expansions in exon 1 of the *HTT* gene. In addition to germline CAG expansions, somatic repeat expansions in neurons also contribute to HD pathogenesis. The DNA mismatch repair gene, MSH3, identified as a genetic modifier of HD onset and progression, promotes somatic CAG expansions, and thus presents a potential therapeutic target. However, what extent of MSH3 protein reduction is needed to attenuate somatic CAG expansions and elicit therapeutic benefits in HD disease models is less clear. In our study, we employed potent di-siRNAs to silence mouse *Msh3* mRNA expression in a dose-dependent manner in Hdh^Q111/+^ mice and correlated somatic *Htt* CAG instability with MSH3 protein levels from simultaneously isolated DNA and protein after siRNA treatment. Our results reveal a linear correlation with a proportionality constant of ~ 1 between the prevention of somatic *Htt* CAG expansions and MSH3 protein expression in vivo, supporting MSH3 as a rate-limiting step in somatic expansions. Intriguingly, despite a 75% reduction in MSH3 protein levels, striatal nuclear HTT aggregates remained unchanged. We also note that evidence for nuclear *Msh3* mRNA that is inaccessible to RNA interference was found, and that MSH6 protein in the striatum was upregulated following MSH3 knockdown in Hdh^Q111/+^ mice. These results provide important clues to address critical questions for the development of therapeutic molecules targeting MSH3 as a potential therapeutic target for HD.

## Introduction

Huntington disease (HD) is a rare autosomal dominant movement disorder characterized by progressive involuntary choreatic movements, as well as cognitive and behavioral disturbances ^1,2^. While neuronal loss in the striatum represents the primary manifestation of HD, the disease affects multiple brain regions, including cortical areas. The underlying cause of HD is an elongated CAG trinucleotide repeat expansion in exon 1 of the *HTT* gene, resulting in the formation of mutant HTT protein with an abnormally long poly-glutamine tail that exhibits a propensity to aggregate ^3,4^. However, the length of germline CAG repeat expansions is not sufficient to trigger neurodegeneration. Additional somatic expansions are required, which occur specifically in the cell types and tissues most affected in HD ^5–7^. Studies in mice have highlighted the crucial role of DNA mismatch repair (MMR) genes in mediating these somatic CAG repeat expansions ^8–10^. Genome-wide association studies have also identified several MMR genes associated with the onset and progression of HD ^11,12^. Among these MMR genes, MSH3 has emerged as a promising therapeutic target for mitigating somatic repeat expansions and potentially slowing HD progression ^13,14^.

MSH3 forms a heterodimer with MSH2, known as MutSβ, which plays a vital role in the DNA mismatch repair process. MutSβ is responsible for detecting DNA mismatch loops and facilitating their subsequent correction by downstream mismatch repair machinery ^15^. However, the mechanism by which MutSβ facilitates somatic repeat expansions remains poorly understood. It is believed that extrahelical DNA extrusions formed by strand-slippage within the CAG repeat tract serve as a substrate for MutSβ. Several models exist to explain how subsequent DNA excisions and resynthesis, in the presence of extrusions, can lead to repeat expansions or contractions ^16^. Importantly, another complex, MutSα, composed of MSH2 and MSH6, exhibits partial overlap in mismatch recognition specificity with MutSβ for repair but not expansions. This overlap may explain the extensive tumorigenesis observed in *Msh2* knockout and *Msh6*/*Msh3* double knockout mice, while *Msh3* knockout alone is well tolerated in mice ^17,18^. In humans, heterozygous mutations in *MSH3* do not present a clear increased risk of cancer while biallelic loss-of-function or predicted loss-of-function mutations are associated with familial colorectal adenomatous polyposis ^19^. In contrast, heterozygous mutations in several other MMR genes, including *MLH1*, *MSH2*, *PMS2*, *MSH6*, and *FAN1*, are linked to cancer ^20–22^. These findings suggest that *MSH3* may represent a HD-linked MMR gene with an acceptable safety range in terms of cancer risk when its expression is reduced.

Previous studies have shown that reducing MSH3 protein expression, through genetic ^9^ or pharmacological manipulation ^23^, effectively prevents somatic CAG repeat expansions. O'Reilly and colleagues successfully utilized di-valent siRNAs (di-siRNAs) to decrease somatic repeat expansions in both Hdh^Q111/+^ and human *Htt* BAC CAG transgenic mice^23^. Di-siRNA’s are a novel siRNA scaffold with excellent distribution and durability in the central nervous system (CNS)^24^. However, prior studies have not investigated the quantitative relationship between MSH3 expression levels and the prevention of somatic instability in detail. Establishing this correlation is crucial for the development of MSH3-targeting drugs and for gaining mechanistic insights into somatic instability. In this study, we systematically analyze *Htt* CAG somatic instability across a wide range of MSH3 protein expression levels, resulting from treatment of Hdh^Q111/+^ mice with varying doses of a di-siRNA targeting the *Msh3* mRNA. Additionally, we explore the impact of pharmacologically reducing somatic repeat expansions on HTT protein accumulation, potentially elucidating further the connection between these two important pathogenic events in HD.

## Materials and methods

### Di-siRNA

A di-siRNA designed against a previously published sequence within the human MSH3 mRNA ^23,24^, was selected to target the identical *Msh3* sequence in mouse. This sequence has no other genome-wide matches in either human or mouse. This di-siRNA was designed, synthesized, and purified to > 90% full-length product at Atalanta Therapeutics, Inc. Test articles and vehicle (PBS) were assessed in a blinded fashion.

### Mice

Cryo-rederived Hdh^Q111/Q111^ mice (Jax strain 00456) were maintained on a mixed CD1/ 129 genetic background by breeding CD1 (Charles River) females with homozygous Hdh^Q111/Q111^ males generating heterozygous mice Hdh^Q111/+^. Genotypes were confirmed from ear biopsies at Laragen Inc. All procedures on the mice were approved by Biogen’s Institutional Animal Care and Use Committee (Cambridge, MA) and carried out in strict accordance with the US National Institutes of Health “Guide for the Care and Use of Laboratory Animals” guidelines ^25^. Further the study was reported in accordance with ARRIVE guidelines 2.0 (https://arriveguidelines.org/arrive-guidelines). Mice were maintained in a temperature-controlled space with a 12 h light/dark cycle. At study end, mice were euthanized via CO2 asphyxiation, before transcardiac perfusion with ice-cold PBS. Cortex, striatum, and cerebellum from the right brain hemisphere and a portion of the median lobe of the liver were collected and immediately frozen on dry ice. The left-brain hemisphere was placed in 10% neutral buffered formalin for 48 h before further processing.

### Treatment of mice with di-siRNA

Heterozygous male and female Hdh^Q111/+^ mice were equally balanced in groups for sex and germline CAG repeat size. Mice were treated by intracerebroventricular delivery (ICV) with either 3 nmol MSH3 di-siRNA, 10 nmol MSH3 di-siRNA, or vehicle (PBS) solution (n = 10 per group). For this procedure, mice were anesthetized with 2% isoflurane and maintained on 0–2% isoflurane throughout. Prophylactic analgesia was provided by 1 mg/kg subcutaneous injection of sustained-release buprenorphine. Approximately 10 min before mice were placed into a stereotaxic apparatus, a 1 cm midline incision was made over the dorsal surface of the skull exposing the bregma. Coordinates used for ICV injection into the lateral right ventricle were − 0.45 mm anteroposterior, − 1.1 mm mediolateral, and − 2.4 mm dorsoventral from dura, relative to bregma. A small hole in the skull was made with a hand-held drill to allow access for a 33-gauge flat-tipped 10 mL Hamilton syringe (701) which was slowly lowered into place and delivered 10 µL of test article at a rate of 1 mL/min. The needle was kept in place for 5 min after the completion of the infusion before being slowly removed. The skin incision was closed with sterile sutures and the mice were placed in a heated recovery chamber kept around 37 °C to recover.

### Protein, mRNA, and DNA isolation from mouse tissue

Protein, mRNA, and DNA were simultaneously isolated from dissected mouse tissue using the AllPrep DNA/RNA/Protein Mini Kit (QIAGEN, 80004) following the manufacturer's provided protocol. The concentrations of RNA and DNA were determined by measuring the absorbance at 260 and 280 nm using a Nanodrop UV–Vis Spectrometer (ThermoFisher). The protein precipitates were resuspended and fully homogenized in 250 µl of RIPA buffer containing 0.5% SDS by a high intensity ultrasonic water bath at 50A for 30 s (QSonica 500 with a cup horn). The lysates were subsequently normalized for protein content using the BCA protein assay (ThermoFisher).

### Determination of somatic CAG repeat expansion index

HTT exon 1, including CAG repeats, was PCR-amplified in a 50 µl reaction using 200 ng of genomic DNA, AmpliTaq Gold 360 Mastermix (Thermofisher), 4% 360 GC enhancer and specific primers. The forward primer, 31329 (ATGAAGGCCTTCGAGTCCCTCAAGTCCTTC), was labeled with 6-FAM, while the reverse primer, 33934 (GGCGGCTGAGGAAGCTGAGGA), was unlabeled ^26^. The polymerase activation and DNA denaturation was carried out at 95 °C for 10 min. Subsequently, the reaction underwent 35 cycles of PCR amplification with denaturation at 95 °C for 30 s, primer annealing at 60 °C for 30 s, extension at 72 °C for 60 s, and a final extension at 72 °C for 7 min. The PCR products, approximately 413 bp for Hdh^Q111/+^, were mixed with formamide and separated using the ABI 3730xl DNA analyzer (Applied Biosystems) along with GeneScan 600 LIZ v2.0 as an internal size standard (Applied Biosystems). The somatic CAG repeat expansion index was determined using the Autogenescan (https://github.com/BranduffMcli/AutoGenescan) and Fragman (Giovanny Covarrubias-Pazaran 2016, BMC Genetics) software packages, applying a peak height selection threshold of > 10% ^27^.

### Western blotting

For protein analysis, the normalized lysates were separated on NuPAGE™ gels (4–12%) under reducing conditions using the MES SDS Running Buffer system. Subsequently, the proteins were transferred to PVDF membranes using the iBlot™ 2 Gel Transfer system. To block non-specific binding, the membranes were incubated in 5% milk in TBS-T (TBS with 0.05% Tween) and then probed with primary antibodies, which were diluted in 5% milk in TBS-T. The primary antibodies used included 1F6 from Millipore Sigma for MSH3, EPR21017-2 from Abcam for MSH2, EPR3945 from Abcam for MSH6, and C4 from Millipore Sigma and 13E5 from Cell Signaling for actin. Bound antibodies were detected using enhanced chemiluminescence (ECL Prime, Cytiva) with a ChemiDoc MP imaging system (BioRad) and quantified by densitometry using Image Lab software 6.1 (BioRad). Each sample was quantified from at least two independent gels (unless indicated otherwise) with different loading orders to minimize bias. Furthermore, all samples from the control group were loaded on each gel for normalization purposes (unless indicated otherwise).

### RT-qPCR

High-Capacity cDNA Reverse Transcription Kit (ThermoFisher) was used to synthesize cDNA from RNA samples. ThermoFisher Taqman qPCR assays were used for MSH3 (Mm00487756_m1), MSH2 (Mm00500563_m1), MSH6 (Mm00487761_m1), and housekeeping gene TBP (Mm01277042_m1). RT-qPCR was performed in a 10 µl reaction using TaqMan Fast Advanced Master Mix for qPCR (ThermoFisher) following manufacturer protocol on QuantStudio 7 Flex (ThermoFisher). Data were normalized to TBP and analyzed via the 2^−ΔΔCT^ method with ThermoFisher Connect Platform and Microsoft Excel.

### Immunofluorescence

Brain hemispheres fixed in neutral buffered formalin were embedded in paraffin and 5 µm serial sections were collected (performed by Inotiv, Boulder, CO). Five sections containing the striatum at approximately 300 µm intervals were stained for DARPP-32 (Abcam ab40801) and Htt (EM48, Millipore Sigma MAB5374) using the Leica Bond autostainer. Briefly, sections were deparaffinized and subjected to heat-induced epitope retrieval using sodium citrate buffer (pH = 6.0). Permeabilization was achieved with 0.1% TX-100 for 30 min, followed by biotin blocking (Abcam ab64212). Following sequential primary antibody incubation for 60 min each, a biotin conjugated goat anti-mouse secondary antibody (Abcam ab6788) followed by Alexa Fluor 647 conjugated streptavidin (ThermoFisher S21374) for EM48 and an Alexa Fluor 488 conjugated goat anti-rabbit antibody (ab150077) for DARPP-32 were used as secondary antibodies. Hoechst nuclear counterstain (Thermo Scientific 62249) was added for 3 min as the final step. Slides were imaged at 20× using the Panoramic P250 slide scanner (3DHISTECH). Regions of interest (ROI) around the striatum were drawn using DARPP-32 reactivity as a reference. Htt positive cells in the striatum were counted by Hoechst co-localization and adaptive thresholding using Visiopharm software. Staining index is calculated as the product of average Htt intensity per cell and the number of Htt positive cells divided by the area of the ROI. DARPP-32 intensity is calculated using Visiopharm as the mean relative fluorescent unit (0–255) of the FITC channel within the striatum normalized to the ROI area (µm^2^). Each data point is the mean of five sections.

### RNAscope

Brain hemispheres fixed in neutral buffered formalin were embedded in paraffin and 5 mm serial sections were collected (performed by Inotiv, Boulder, CO). Sections were stained for MSH3 (Advanced Cell Diagnostics 833309) using RNAscope^®^ VS Universal HRP Reagent Kit (Advanced Cell Diagnostics 323200) on Ventana Discovery Ultra autostainer.

Sections were counterstained with 10 µg/mL Hoechst 33342 (ThermoFisher H3570) for 5 min. Slides were coverslipped with ProLong™ Gold Antifade Mountant (ThermoFisher P36930) and imaged at 40× confocal on the TissueGnostics TissueFAXS SL scanning system.

### Statistical analysis

Analyses were performed with GraphPad Prism 9.3.1. Significant level was defined as p < 0.05. Intergroup differences were tested using Student’s t-test and one-way ANOVA. Multicomparisons were corrected with Tukey’s post hoc test when comparing three or more groups. Data were displayed showing error bars with SDs. Correlation analysis was done with a simple linear regression model. Data were displayed showing 95% confidence interval and R^2^.

## Results

### Study design for investigating quantitative relationship between MSH3 expression and somatic instability in Hdh^Q111/+^ mice

Motivated by the need to elucidate the quantitative relationship between MSH3 expression and the prevention of somatic instability, we conducted an in-depth investigation aiming to correlate somatic instability with a wide range of MSH3 protein expression levels in an HD mouse model exhibiting somatic CAG repeat expansions. To accomplish this, our experimental approach involved the dose-dependent repression of MSH3 in Hdh^Q111/+^ mice using ICV of an MSH3 di-siRNA. Prior to this experiment, we performed a small dose finding study in wild-type mice demonstrating a dose-dependent reduction of MSH3 protein upon ICV injection of 0.5 to 20 nmol MSH3 di-siRNA (see Supplementary Fig. 1 and Supplementary Material and methods). We next confirmed that our in-house cohort of Hdh^Q111/+^ mice displayed age-dependent CAG somatic expansions in *Htt*. Measurements of the somatic instability index at 2, 3, 6, and 12 months of age in various brain and peripheral tissues from these mice demonstrated high somatic instability in the striatum and liver, and to a lesser extent in the cortex, cerebellum, spleen, and tail (Supplementary Fig. 2A–F) validating previous findings ^28^. Notably, there was a > twofold increase in the somatic expansion index between 3 and 6 months of age in the striatum, suggesting an optimal assay window for a 3-month treatment study starting at 3 months of age (Supplementary Fig. 2F).

**Figure 1:**
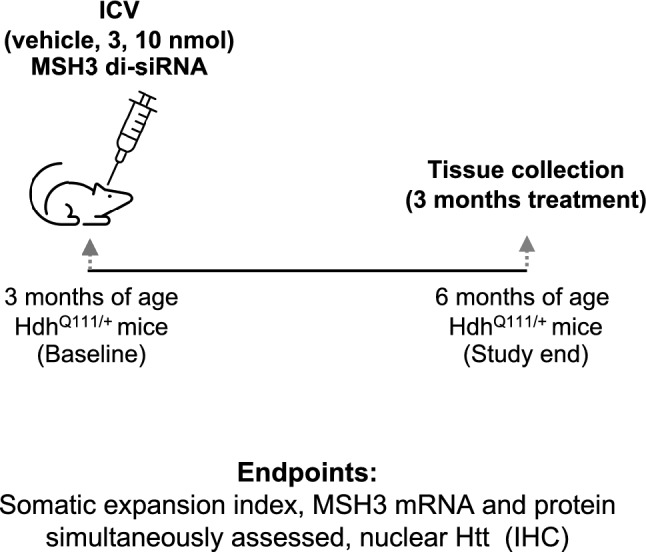
Study design for investigating quantitative relationship between MSH3 expression and somatic instability in Hdh^Q111/+^ mice treated with MSH3 di-siRNA.

Consistent with these observations, we treated 3-month-old Hdh^Q111/+^ mice with ICV delivery of vehicle, 3 nmol MSH3 di-siRNA, and 10 nmol MSH3 di-siRNA. These dosages were selected based on findings from the dose finding study (see Supplementary Fig. 1) as well as prior experience with di-siRNAs. Three months after di-siRNA administration, we collected protein, mRNA, and DNA simultaneously from brain and peripheral tissue samples, enabling diverse correlation analyses. Tissue was also collected from untreated 3-month-old mice (baseline) and 6-month-old mice (see Supplementary Fig. 2). These naïve animals were from the same matings as the treated animals and tissues were all processed in parallel. This design (Fig. 1) allows for a quantitative investigation of the correlation between MSH3 reduction and somatic instability.

### Dose-dependent reduction of MSH3 protein and mRNA by silencing MSH3 using a di-siRNA in Hdh^Q111/+^ mice

**Figure 2:**
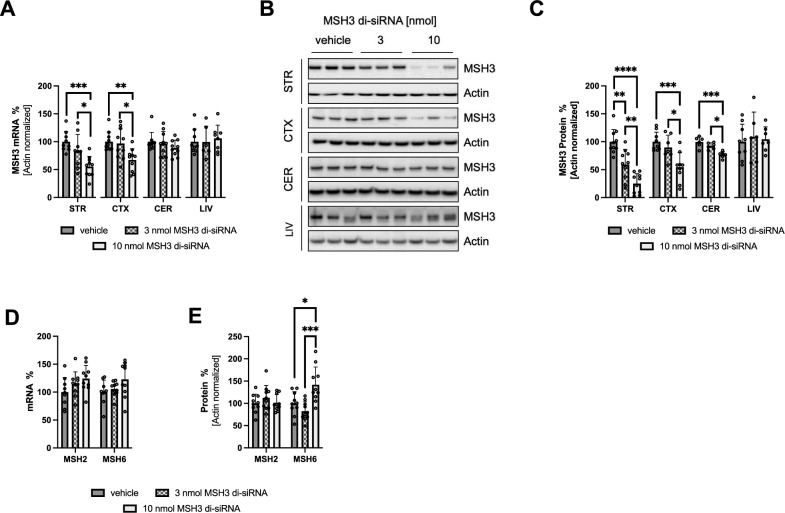
Dose-dependent repression of MSH3 expression in Hdh^Q111/+^ mice following ICV MSH3 di-siRNA treatment. (**A**) RT-qPCR analysis of *Msh3* mRNA levels in the striatum (STR), cortex (CTX), cerebellum (CER), and liver (LIV). (**B**) Representative Western blots and (**C**) quantitative analysis of MSH3 protein using densitometry in the striatum, cortex, cerebellum, and liver. Each sample has been analyzed on multiple independent blots. (**D**) RT-qPCR analysis of *Msh6* and *Msh2* mRNA levels in the striatum (**E**) quantitative analysis of MSH6 and MSH2 protein using densitometry in the striatum. Protein levels were normalized to β-actin and expressed as percentage of vehicle-treated mice. Error bars represent mean ± SD; n = 6–10 per group. One-Way ANOVA with Tukey’s post hoc analysis, *p < 0.05, **p < 0.01, ***p < 0.001 and ****p < 0.0001.

To confirm the dose-dependent repression of MSH3 expression in Hdh^Q111/+^ mice following a 3-month treatment with 3 and 10 nmol of MSH3 di-siRNA, we conducted measurements of *Msh3* mRNA and MSH3 protein levels using RT-qPCR and semi-quantitative Western blotting, respectively. These measurements were performed in the striatum, cortex, cerebellum, and liver. As depicted in Fig. 2A, we observed a significant dose-dependent reduction in *Msh3* mRNA levels in the striatum (100 ± 18% for vehicle, 83 ± 30% for 3 nmol, and 55 ± 18% for 10 nmol di-siRNA; n = 9–10 per group; mean ± SD) and cortex (100 ± 17% for vehicle, 97 ± 28% for 3 nmol, and 67 ± 20% for 10 nmol di-siRNA; n = 10 per group). A slight numerical reduction in *Msh3* mRNA was also noted in the cerebellum. Furthermore, we demonstrated a dose-dependent decrease in MSH3 protein levels in the striatum (100 ± 22% for vehicle, 59 ± 27% for 3 nmol, and 25 ± 18% for 10 nmol di-siRNA; n = 10), cortex (100 ± 17% for vehicle, 90 ± 22% for 3 nmol, and 54 ± 26% for 10 nmol di-siRNA; n = 7–9), and cerebellum (100 ± 9% for vehicle, 92 ± 6% for 3 nmol, and 77 ± 6% for 10 nmol di-siRNA; n = 6), as shown in Fig. 2B and C and Supplementary Figs. 5–7 and Supplementary Tables 1–3. Notably, no reductions in *Msh3* mRNA and MSH3 protein levels were observed in the liver, as the di-siRNA was directly administered into the CNS (Fig. 2A–C, Supplementary Fig. 8 and Supplementary Table 4). The reduction of MSH3 also did not affect the levels of *Msh2* and *Msh6* mRNA in the striatum (Fig. 2D), consistent with a previous report (O’Reilly et al., 2023). However, striatal MSH6 protein is significantly (p < 0.05) upregulated upon 10 nmol MSH3 di-siRNA treatment (Fig. 2E, Supplementary Fig. 9 and Supplementary Table 5; 100 ± 26% for vehicle, 82 ± 21% for 3 nmol, and 142 ± 40% for 10 nmol di-siRNA; n = 10). It is noteworthy that there is a significant (p < 0.01) negative linear correlation between the somatic expansion index and MSH6 protein in the 10 nmol treatment group (Supplementary Fig. 3A). No changes in MSH2 protein were observed (Fig. 2E, Supplementary Fig. 10 and Supplementary Table 6). Additionally, a subset of mice treated with 10 nmol MSH3 di-siRNA was sacrificed after only 2 months of treatment. These mice exhibited a 71.5 ± 20.0% reduction in striatal MSH3 protein and a 40.4 ± 13.0% reduction in *Msh3* mRNA compared to baseline (Supplementary Figs. 4A–C and 5, Supplementary Table 1). This is very similar to the knockdown observed at the end of the 3 months treatment, indicating a constant knockdown over the 3 months duration of the study. Collectively, these findings demonstrate a dose-dependent reduction of MSH3 upon treatment with the *Msh3* mRNA targeted di-siRNA.

### Unveiling an inaccessible nuclear MSH3 mRNA pool resistant to knockdown by di-siRNA

**Figure 3:**
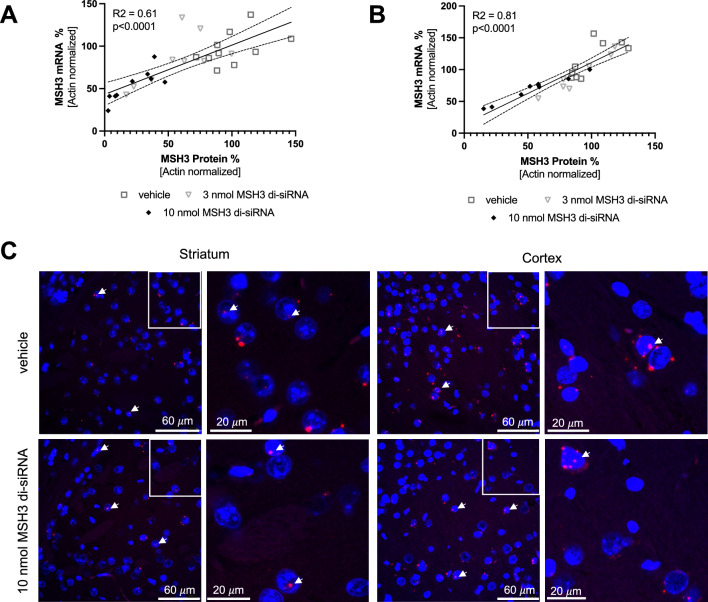
Nuclear *Msh3* mRNA pool that is not accessible by MSH3 di-siRNA. (**A**) The graph demonstrates the linear correlation between striatal MSH3 mRNA and protein levels in Hdh^Q111/+^ mice post 10 nmol MSH3 di-siRNA treatment. The correlation coefficient (R^2^) is 0.61, with a Y-intercept at 43.2 and a 95% confidence interval ranging from 29.8 to 56.6 (p < 0.001). (**B**) The linear correlation between cortical *Msh3* mRNA and protein levels is depicted, presenting a correlation coefficient (R^2^) of 0.81, a Y-intercept at 14.0, and a 95% confidence interval from − 3.7 to 31.6 (p < 0.001). (**C**) Msh3 mRNA (red) localization in striatum and cortex detected with RNAscope post vehicle and 10 nmol MSH3 di-siRNA treatment. Nuclei stained with Hoechst 33342 (blue). Arrows indicate nuclei with nuclear Msh3 mRNA. The second and fourth columns display higher magnification views of the regions outlined by white boxes in the first and third columns, respectively.

Upon comparing striatal and cortical Msh3 mRNA levels with their corresponding protein levels, we identified a linear correlation, substantiating our quantification methods. The striatal mRNA/protein Y-intercept was approximately 43% (with a 95% confidence interval of 30–57%, Fig. 3A) and the cortical samples had a Y intercept of 14% (with a 95% confidence interval of − 4 to 32%, Fig. 3B). These intercepts highlight a disconnect between RNA and protein knockdown levels. To explore this, we used RNAscope to visualize Msh3 transcript localization in the striatum and cortex. We observed a significant pool of Msh3 mRNA in the nucleus (Fig. 3C), which is likely inaccessible to translational machinery and RNA interference. This can account for the observed disconnect between RNA and protein knockdown levels.

### Dose-dependent reduction of somatic repeat expansion index by silencing MSH3 using a di-siRNA in Hdh^Q111/+^ mice

**Figure 4:**
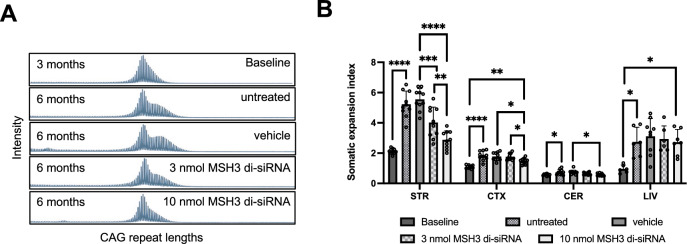
Dose-dependent reduction of somatic repeat expansion index by silencing *Msh3* using di-siRNA in Hdh^Q111/+^ mice. (**A**) Representative fragment analysis traces showing somatic CAG repeat expansions in the striatum of Hdh^Q111/+^ at 3 months of age, and untreated, treated ICV with vehicle, 3 nmol di-siRNA, and 10 nmol di-siRNA at 6 months of age. (**B**) Quantification of the somatic expansion index in the striatum (STR), cortex (CTX), cerebellum (CEB) and liver (LIV). Error bars represent mean ± SD; n = 10. One-way ANOVA with Tukey’s post hoc analysis, *p < 0.05, **p < 0.01, ***p < 0.0001 and ****p < 0.0001.

We evaluated the impact of MSH3 di-siRNA treatment on somatic CAG repeat expansions in *Htt* in brain and peripheral tissues of Hdh^Q111/+^ mice. Somatic CAG repeat expansions were quantified from fragment analysis traces by determining a somatic expansion index (SEI) ^27^. As depicted in Fig. 4A and B, administration of 3 and 10 nmol MSH3 di-siRNA resulted in a reduction of striatal somatic CAG repeat expansions by 45.1 ± 29.4% and 78.1 ± 17.3%, respectively, compared to baseline animals and vehicle-treated animals (3 months baseline: 2.1 ± 0.2; untreated at study end: 5.3 ± 0.9; vehicle-treated mice at study end: 5.6 ± 0.7; treated with 3 nmol di-siRNA at study end: 4.0 ± 1.0; treated with 10 nmol di-siRNA at study end: 2.9 ± 0.6 SEI; mean ± SD, n = 10). Moreover, a significant dose-dependent treatment effect was observed in the cortex (3 months baseline: 1.1 ± 0.11; untreated at study end: 1.8 ± 0.31; vehicle-treated mice at study end: 1.8 ± 0.27; treated with 3 nmol di-siRNA at study end: 1.8 ± 0.21; treated with 10 nmol di-siRNA at study end: 1.5 ± 0.2 SEI; mean ± SD, n = 10) and cerebellum as well (3 months baseline: 0.58 ± 0.07; untreated study end: 0.74 ± 0.18; vehicle-treated mice at study end: 0.73 ± 0.15; treated with 3 nmol di-siRNA at study end: 0.64 ± 0.08; treated with 10 nmol di-siRNA at study end: 0.58 ± 0.08 SEI; mean ± SD, n = 10) (Fig. 4B). Notably, somatic expansions in a peripheral tissue such as the liver were not prevented, consistent with the CNS-specific route of di-siRNA administration (Fig. 4B). The subset of mice treated with 10 nmol MSH3 di-siRNA for only 2 months did show an almost complete prevention of striatal somatic repeat CAG expansions (Supplementary Fig. 4D and E). The SEI’s after 2- or 3-months treatment with 10 nmol are not statistically different (not shown). Collectively, these findings demonstrate that MSH3 di-siRNA treatment effectively prevents the formation of somatic CAG repeat expansions in a dose-dependent manner in Hdh^Q111^^/+^ mice.

### Prevention of somatic CAG repeat expansion is directly proportional to MSH3 protein reduction.

**Figure 5:**
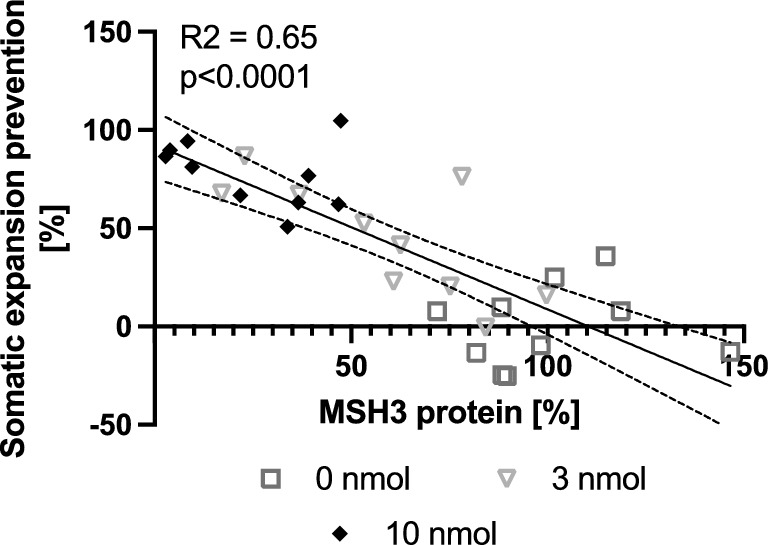
Prevention of somatic CAG repeat expansion is directly proportional to MSH3 protein reduction. Graph representing the linear correlation between prevention of somatic expansions and protein levels in striatal tissue. The correlation coefficient (R^2^) is 0.65, with a Y intercept of 92.4 and a 95% confidence interval of 75.4 to 109.5 and with x intercept of 110.4 and a 95% confidence interval of 96.0 to 133.4, p < 0.0001.

To investigate the quantitative association between MSH3 expression and somatic instability, we generated a plot of striatal MSH3 protein levels against the observed inhibition of somatic expansions in the striatum (Fig. 5). Our analysis utilizing linear regression (R^2^ = 0.65, p < 0.0001), revealed a clear directly proportional relationship between MSH3 protein levels and the prevention of somatic CAG repeat expansions in Hdh^Q111/+^ mice, with a proportionality constant of approximately 1. These findings provide evidence supporting that MSH3 activity plays a critical role in the rate-limiting step of somatic CAG repeat expansion formation in vivo.

### No effect of MSH3 di-siRNA treatment on nuclear Htt staining in Hdh^Q111/+^ mice

**Figure 6:**
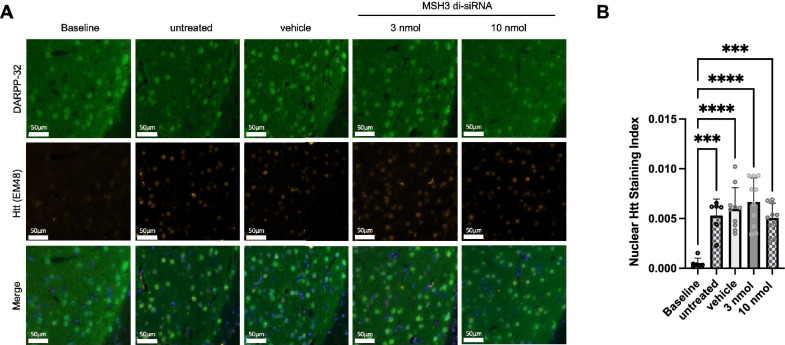
No Effect of MSH3 di-siRNA treatment on nuclear HTT staining in Hdh^Q111/+^ mice. (**A**) Representative immunofluorescence images showing Alexa Fluor 647 labelled HTT nuclear inclusions (EM48), Alexa Fluor 488 labelled DARP-32 and Hoechst staining in striatal sections of Hdh^Q111/+^ mice at 3 months of age, untreated, treated with vehicle, 3 nmol di-siRNA, and 10 nmol di-siRNA at 6 months of age. (**B**) Quantification of the nuclear HTT staining index in the striatum. Error bars represent mean ± SD; n = 6–10. One-way ANOVA with Tukey’s post hoc analysis, ***p < 0.0001 and ****p < 0.0001.

Following the successful reduction of CAG somatic repeat expansions in *Htt* through MSH3 di-siRNA treatment in Hdh^Q111/+^ mice, we sought to investigate the potential implications of this phenomenon on downstream pathogenesis. Notably, crossing Hdh^Q111/+^ mice with *Msh2* or *Msh3* deficient mice results in a decrease in nuclear Htt accumulation^8,9^. To determine if our pharmacological intervention targeting MSH3 could reproduce these findings, we conducted immunofluorescence analysis of nuclear HTT in striatal sections obtained from our treatment study (Fig. 6). We observed a significant increase in nuclear HTT staining in untreated and vehicle treated Hdh^Q111/+^ mice at the study's conclusion compared to the mice at the beginning of the treatment (Fig. 6A and B). This observation indicates the existence of an appropriate assay window for detecting treatment effects. However, treatment of Hdh^Q111/+^ mice with 3 or 10 nmol of MSH3 di-siRNA did not induce any significant alterations in the nuclear HTT staining index when compared to vehicle-treated or untreated mice (Fig. 6A and B). Consequently, these findings raise questions regarding the involvement of somatic CAG repeat instability in Htt aggregation in Hdh^Q111/+^ mice above 3 months of age.

## Discussion

Our study presents strong evidence for a direct correlation between somatic instability of the CAG repeat within Htt Exon 1 and the levels of pharmacologically manipulated MSH3 in an HD mouse model. The observed proportionality constant of approximately 1 supports the prior hypothesis that the binding of MutSβ to DNA loops formed by slipped CAG repeats is a rate-limiting step in the somatic expansion process. These findings have implications for the development of therapeutic interventions targeting MSH3 in HD and other triplet repeat disorders characterized by somatic instability. According to our results, achieving a complete cessation of somatic expansions would require an almost 100% reduction in MSH3 levels. However, such a substantial reduction may not necessarily be achievable in human subjects. Nonetheless, reducing MSH3 levels by 50%, a reduction considered safe based on genetic studies, could lead to a corresponding 50% decrease in somatic instability. Our pharmacology study corroborates and expands upon the findings of previous genetic mouse studies ^9,29^. It is noteworthy that all our correlation analyses benefited greatly from the simultaneous isolation of DNA and protein from the same tissue samples, making this approach valuable for future similar experiments. Importantly, despite a significant reduction in the MSH3 protein and somatic expansion index, observed to be 75 ± 18% and 78 ± 17% respectively in the 10 nmol di-siRNA group, there was no evidence of contractions in somatic repeat expansions. Notably, even the two mice that demonstrated over 95% striatal MSH3 knockdown did not display lower somatic repeat expansions than those found in mice at study start. Thus, our data suggests that pharmacological reduction of MSH3 activity can effectively prevent further expansions but may not significantly reduce existing expansions.

Our study did not allow us to determine the specific degree of somatic expansion reduction necessary to achieve therapeutic benefits. For instance, we did not find any evidence of neurodegeneration, as assessed by DARP32 staining, between 3 and 6 months of age in our animals (Supplementary Fig. 11). This finding diverges from a previous report in this mouse model ^30^ but is consistent with another study that reported only a marginal decrease in DARP32 staining in mice aged 12 months beyond the animals in our study ^31^. To address in a preclinical setting the degree of somatic expansion reduction to achieve a therapeutic benefit, the utilization of BAC CAG mice, which display somatic instability-dependent HD-like phenotypes at later stages of life ^32^, may be more appropriate. However, we did successfully demonstrate a clear increase in nuclear HTT accumulations between 3 and 6 months of age in Hdh^Q111/+^ mice. Surprisingly, our treatment did not impact these nuclear HTT protein accumulations, contradicting previous reports that suggest that the knockout of *Msh3* or other DNA mismatch repair genes in Hdh^Q111/+^ mice reduces nuclear HTT protein accumulations ^8,9^. Considering that even Hdh^Q111/+^ mice with a heterozygous *Msh3* KO exhibit a significant reduction in accumulations, the pharmacological 75% reduction in MSH3 protein in our study should be sufficient to replicate the genetic findings. One possible explanation for this discrepancy is that the somatic expansions observed between birth and 3 months of age triggered the HTT accumulation process beyond a threshold that cannot be rescued solely by reducing somatic repeat expansions. Another possibility is that the genetic knockout of *Msh3* leads to compensatory mechanisms that affect HTT protein accumulations, such as a reduction in total HTT protein expression. Similarly, our Hdh^Q111/+^ strain may express higher levels of HTT, leading to somatic repeat expansion-independent accumulation of HTT. Therefore, further investigations are warranted to explore the relationship between somatic CAG repeat expansions and the rate of HTT accumulation in preclinical models. Analyzing MSH3 expression in postmortem brains of HD patients carrying *Msh3* variants associated with disease progression hastening or slowing may provide more meaningful insights than relying solely on preclinical models to determine the extent of reduction in somatic CAG repeat expansion, and consequently MSH3 expression, required to achieve therapeutic benefits.

Similar to what has been reported for Htt mRNA ^33^, our data indicates that a significant portion of MSH3 mRNA is resistant to RNA interference since it is retained in the nucleus. The function of this potential nuclear mRNA pool remains unknown, and unlike nuclear *HTT* mRNA ^34,35^, there is no evidence implicating *MSH3* mRNA itself in the disease process. Therefore, this resistant *Msh3* mRNA pool does not pose a concern for the efficacy of RNA interference-based therapeutic approaches targeting *MSH3*. However, it does have implications for PK/PD studies of therapeutic siRNAs targeting *MSH3*. Specifically, measuring MSH3 protein levels is more suitable for assessing the potency of siRNAs targeting MSH3 than measuring MSH3 mRNA levels.

Clinical safety remains a paramount concern when targeting MSH3, given its integral role in the DNA mismatch repair mechanism. By confining MSH3 knockdown to the CNS via intrathecal administration of di-siRNA in humans, we can mitigate potential risks tied to MutSβ activity loss in peripheral tissues, including the colon. Additionally, our findings suggest that MSH6—and, by extension, likely MutSα—could be upregulated via a post-transcriptional compensation mechanism to offset significant MutSβ loss, as observed in the group treated with 10 nmol MSH3 di-siRNA. This discovery is indeed promising and can be explained by how MSH3 and MSH6 compete for stabilization by binding to the common partner protein MSH2, which is well-known in the field ^15^. However, further investigations are necessary to elucidate whether different cell types exhibit unique responses to the knockdown.

There are several technical limitations in our study worth mentioning. Firstly, we opted to exclude a non-targeting control di-siRNA because such a control might introduce its own unique adverse effects. Consequently, we are unable to conclusively determine whether the impacts we noted, like those on somatic repeat instability, are a result of the di-siRNA modality itself or the targeted suppression of MSH3. Secondly, while biochemical techniques for quantitatively measuring Htt aggregates are now well established ^36^, the protein isolation procedure we employed is not compatible with these methods. This incompatibility hinders us from substantiating more the absence of influence on HTT aggregation following the acute reduction of MSH3 in HdhQ111/+ mice. Finally, the noted increase in MSH6 protein levels warrants additional verification and scrutiny. The apparent positive correlation between MSH6 protein and mRNA levels, as indicated in Supplementary Fig. 3B, suggests that the utilized antibody accurately measures MSH6 protein. However, confirmation using an antibody validated by knockout experiments or by employing various MSH6-specific antibodies would provide stronger assurance of the results. All these limitations should be considered in future research studies of a similar nature.

Our findings provide valuable support and guidance for the further development of di-siRNAs targeting MSH3 as a potential therapeutic approach for HD disease. It is important to note that all our experiments were conducted using a mouse model expressing a CAG repeat length (~ 111) that is only found in a small subset of HD patients with childhood onset of the disease. Therefore, it is essential to conduct future experiments to determine whether the quantitative relationship between MSH3 protein levels and somatic CAG repeat expansions, as reported here, holds true for germline CAG repeat lengths (~ 40–50) typically observed in the more common adult-onset form of HD. Additionally, investigations are warranted to examine whether these findings extend to very long CTG repeats found in diseases such as DM1. These follow-up studies will provide further insights into the broader applicability and potential efficacy of targeting MSH3 in different contexts of CAG repeat-associated disorders.

### Supplementary Information

Supplementary Information.

## Data Availability

All data generated or analyzed during this study are included in this published article (and its Supplementary Information file).
