# Supplementary material for: Dose-dependent reduction of somatic expansions but not Htt aggregates by di-valent siRNA-mediated silencing of MSH3 in HdhQ111 mice
Source: Sci Rep. 2024 Jan 24;14:2061. doi: 10.1038/s41598-024-52667-3 (PMC10808119; doi:10.1038/s41598-024-52667-3)
Supplement: Supplementary file 1 — Supplementary Information. [file 41598_2024_52667_MOESM1_ESM.pdf]

Supplementary Data

Dose-dependent reduction of somatic expansions but not Htt aggregates by di-valent siRNA-mediated silencing of MSH3 in HdhQ111 mice

Rachelle Driscoll[1,#], Lucas Hampton[2,#], Neeta A. Abraham[2,#], J. Douglas Larigan[3], Nadine F. Joseph[2], Juan C. Hernandez-Vega[2], Sarah Geisler[1], Fu-Chia Yang[1], Matthew Deninger[4], David T. Tran[4], Natasha Khatri[4], Bruno M.D.C. Godinho[4], Garth A. Kinberger[4], Daniel R. Montagna[2], Warren D. Hirst[2], Catherine L. Guardado[2], Kelly E. Glajch[2], H. Moore Arnold[1], Corrie L. Gallant-Behm[4], Andreas Weihofen[2,*]

[1] Translational Sciences, Biogen, 225 Binney Street, Cambridge MA 02142, USA
[2] Neurodegenerative Diseases Research Unit, Biogen, 225 Binney Street, Cambridge MA 02142, USA
[3] Biotherapeutics and Medicinal Sciences, Biogen, 225 Binney Street, Cambridge MA 02142, USA
[4] Atalanta Therapeutics, 51 Sleeper Street, Boston MA, 02210, USA
[#] contributed equally
* Corresponding author at: Biogen, 225 Binney Street, Cambridge MA 02142, USA; E-mail addresses: andreas.weihofen@biogen.com (A.W)

Supplementary Figures 1-10, Tables 1-6 and Material & Methods:

Supplementary Figure 1

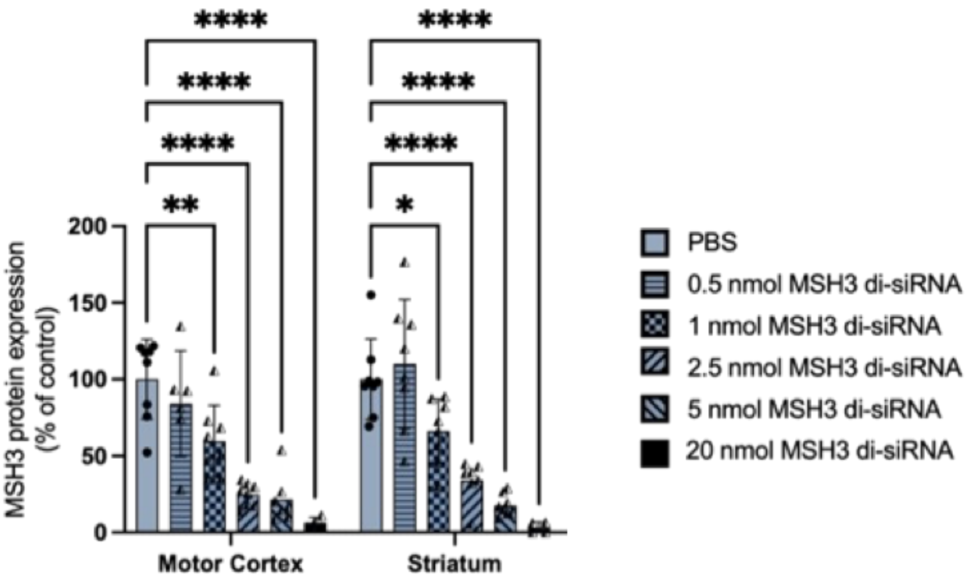

Supplementary Figure 1 Dose-dependent reduction in MSH3 protein in FVB/N mice following ICV MSH3 di-siRNA treatment Quantitative analysis of MSH3 protein using JESS analysis. Protein levels were normalized to β-actin and expressed as percentage of vehicle-treated mice. Error bars represent mean ± SD; n=7 per group. One-Way ANOVA with Tukey's post hoc analysis, * $p<0.05$, ** $p<0.01$, and **** $p<0.0001$.

Supplementary Figure 2

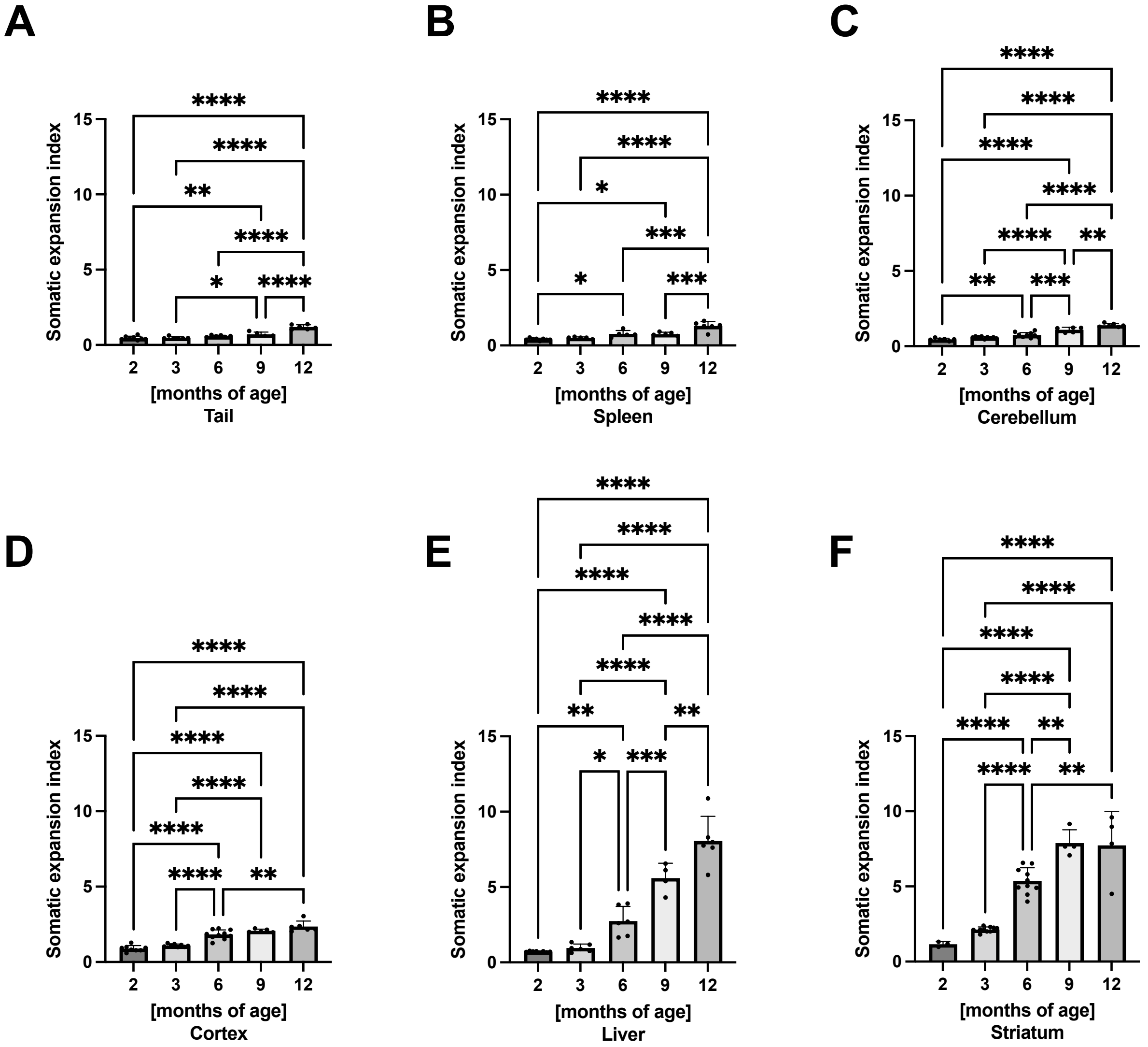

Supplementary Figure 2 Age-dependent increase in somatic CAG repeat expansions in tissues of Hdh^{Q111}/$^{+}$ mice Somatic expansion index in the (A) tail, (B) spleen, (C) cerebellum, (D) cortex, (E) liver and (F) striatum over 2, 3, 6, 9 and 12 months of age. Error bars represent mean ± SD; n=3-10 per age and tissue. One-Way ANOVA with Tukey's post hoc analysis, * p<0.05, ** p<0.01, *** p<0.0001 and **** p<0.0001.

Supplementary Figure 3

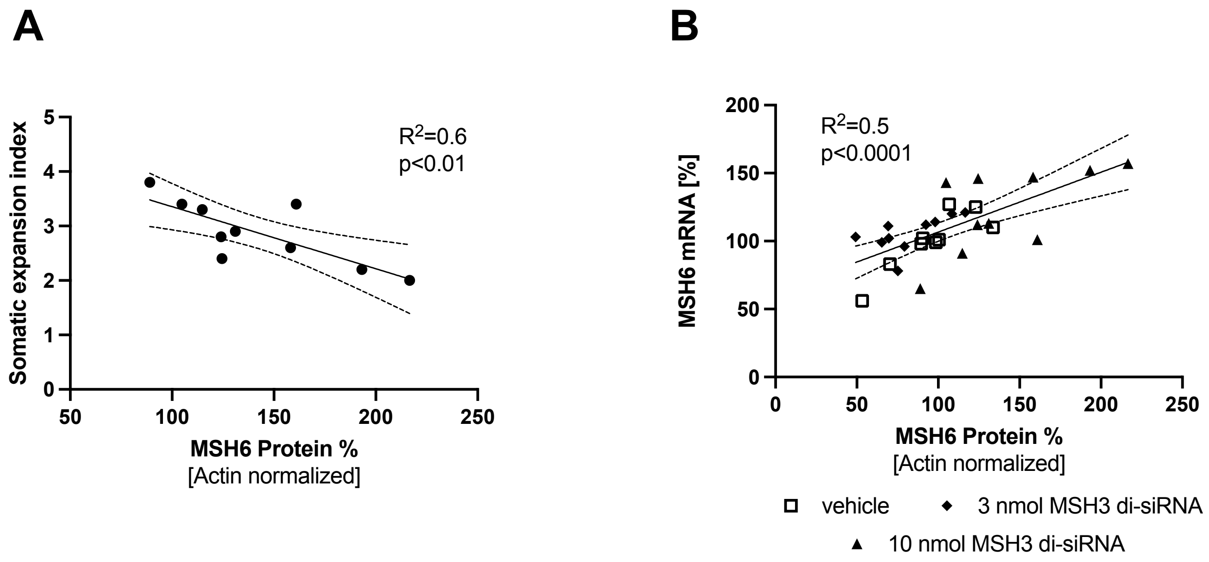

Supplementary Figure 3 A Negative linear correlation between somatic expansion index and MSH6 protein levels in the striatum of HdhQ111/+ mice treated with 10 nmol MSH3 di-siRNA for 3 months **B** Significant positive correlation between MSH6 mRNA and protein in the striatum of $Hdh^{Q111}/^{+}$ mice treated with vehicle, 3 or 10 nmol MSH3 di-siRNA for 3 months. Dashed lines indicate 95% confidence interval.

Supplementary Figure 4

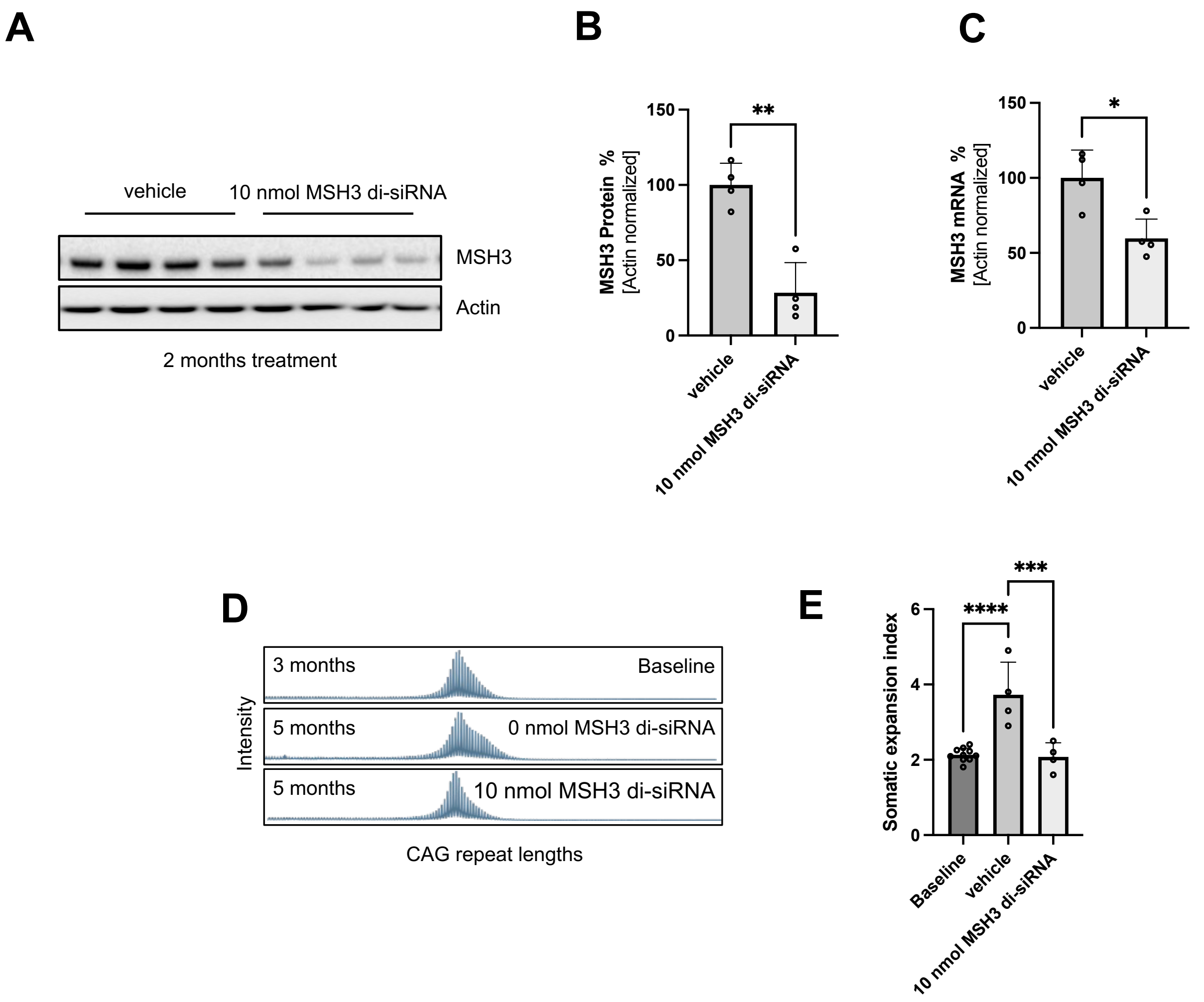

Supplementary Figure 4 Effect of MSH3 di-siRNA treatment on striatal MSH3 expression and somatic instability index in Hdh$^{Q111/+}$ mice after 2 months. (A) Representative Western blots and (B) quantitative analysis of MSH3 protein levels using densitometry. Protein levels were normalized to β-actin and expressed as percentage of vehicle-treated mice. (C) RT-qPCR analysis of MSH3 mRNA. Error bars represent mean ± SD; n=4 per group, Student's t-test, * $p<0.05$, ** $p<0.01$ (D) Representative fragment analysis traces showing somatic CAG repeat expansions and (E) quantification of the somatic expansion index in the striatum of Hdh$^{Q111/+}$ at 3 months and 5 months of age, treated with either vehicle or 10 nmol di-siRNA. Error bars represent mean ± SD; n=4-10 per group. One Way ANOVA with Tukey's post hoc analysis, *** $p<0.001$, **** $p<0.0001$.

Supplementary Figure 5

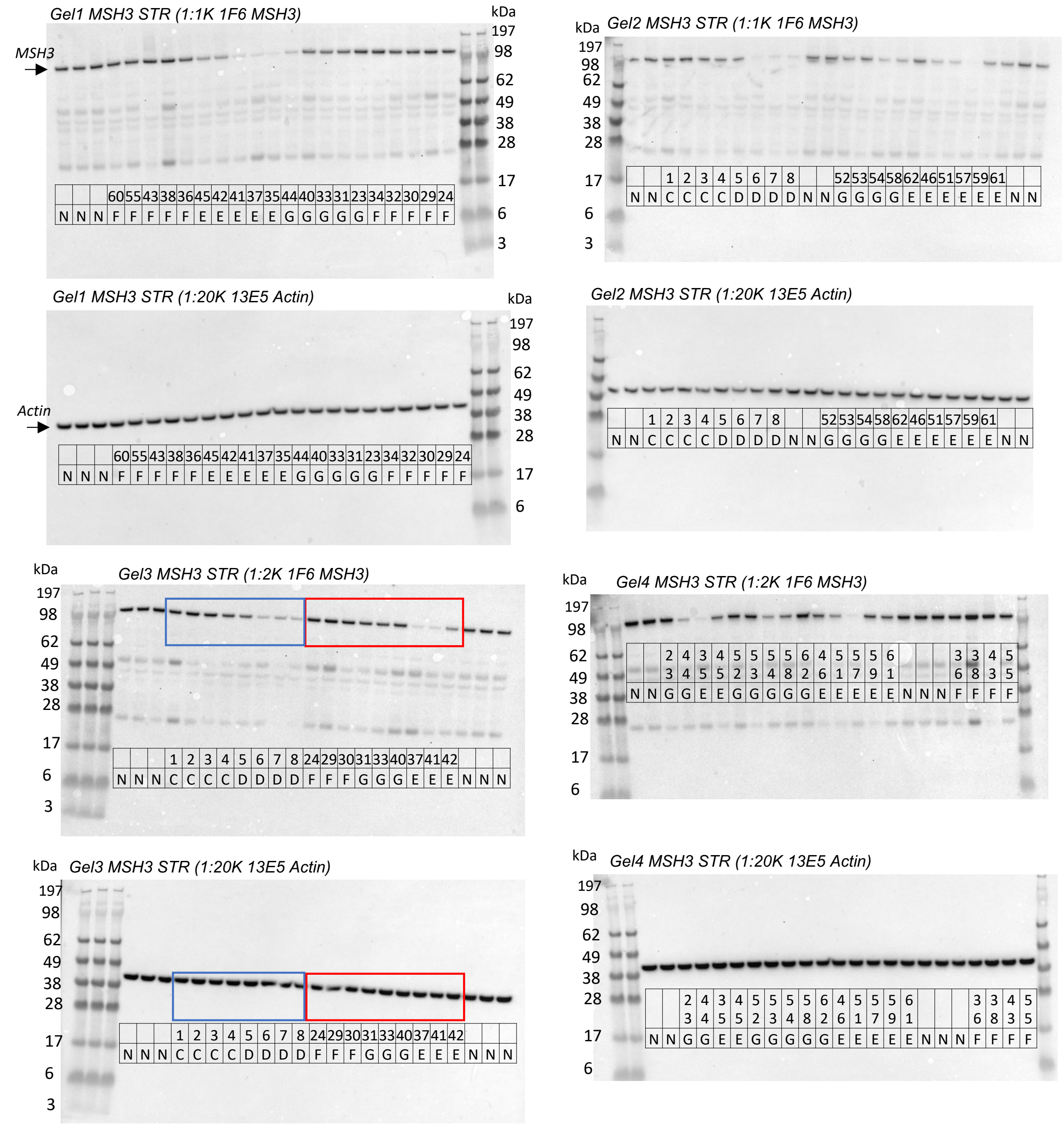

Supplementary Figure 5 Western blot analysis for quantification of striatal MSH3 levels in Hdh$^{Q111/+}$ mice following treatment with vehicle, 3 and 10 nmol MSH3 di-siRNA for 2 or 3 months. Four gels were probed with antibodies 1F6 (MSH3) and 13E5 (actin). Groups: C vehicle and D 10 MSH3 di-siRNA nmol for 2 months (see Supplementary Figure 4A and B) and F vehicle, G 3 nmol and E 10 nmol MSH3 di-siRNA for 3 months (see Figure 2A and B). N is a pool of all F samples, which was used for normalization. Membranes were first probed for MSH3, then for actin after stripping. Blue and red boxes indicate blots used for Supplementary Figure 4A and Figure 2B STR, respectively.

Supplementary Table 1 Striatal MSH3 protein levels Quantifications

Group	Striatum ID	Gel1	Gel2	Gel 3	Gel 4	Average	Average % normalized to F
			% normalized to N				
C	1		122.3	100.1		111.2	93.9
C	2		140.8	105.4		123.1	104.0
C	3		93.9	110.2		102.0	86.1
C	4		98.0	75.8		86.9	73.4
D	5		67.7	54.1		60.9	51.4
D	6		6.8	20.8		13.8	11.6
D	7		12.6	39.5		26.1	22.0
D	8		10.5	29.1		19.8	16.7
E	35	6.5			2.8	4.6	3.9
E	37	11.2		8.6		9.9	8.4
E	41	12.6		9.9		11.2	9.5
E	42	42.1		37.9		40.0	33.8
E	45	50.4			36.1	43.2	36.5
E	46		36.0		56.5	46.3	39.1
E	51		25.7		26.0	25.8	21.8
E	57		3.7		2.8	3.3	2.8
E	59		54.4		56.4	55.4	46.8
E	61		62.2		50.0	56.1	47.4
F	24	137.1		143.8		140.4	118.6
F	29	139.9		207.0		173.5	146.5
F	30	108.1		124.7		116.4	98.3
F	32	106.1				106.1	89.6
F	34	136.0				136.0	114.8
F	36	106.2			87.9	97.0	81.9
F	38	122.2			118.8	120.5	101.8
F	43	116.0			93.6	104.8	88.5
F	55	107.9			62.3	85.1	71.9
F	60	104.5				104.5	88.3
G	23	147.4			88.8	118.1	99.7
G	31	94.5		90.8		92.6	78.2
G	33	80.6		67.6		74.1	62.5
G	40	91.1		87.0		89.1	75.2
G	44	25.2			15.1	20.2	17.0
G	52		66.3		60.1	63.2	53.3
G	53		81.4		62.6	72.0	60.8
G	54		30.0		24.2	27.1	22.9
G	58		45.1		42.1	43.6	36.8
G	62		92.4		107.0	99.7	84.2
N		104.7	76.5	112.5	98.4		
N		95.1	89.2	110.8	89.0		
N		100.1	122.4	94.1	102.7		
N			101.9	96.3	96.3		
N			102.5	79.0	113.7		
N			107.5	107.3			

See Supplementary Figure 5 for corresponding Western blots

Supplementary Figure 6

Gel1 MSH3 CTX (1:1K 1F6 MSH3)

26	30	51	56	27	31	47	48	25	28	29	33	35	36	37	38	39	49	54	58	34	46	53	55	57
F	F	F	F	G	G	G	G	E	E	E	E	B	B	B	B	B	G	G	G	E	E	E	E	E

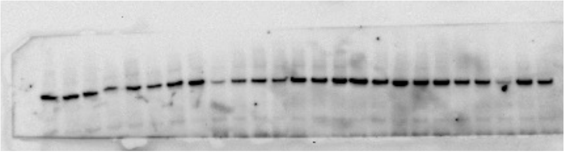

Gel1 MSH3 CTX (1:20K 13E5 Actin)

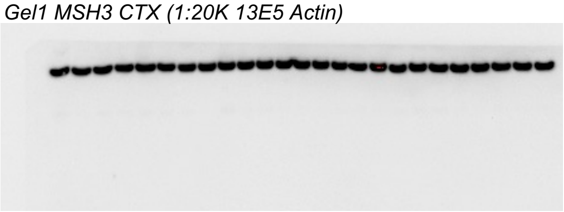

Gel2 MSH3 CTX (1:1K 1F6 MSH3)

25	26	27	28	29	30	31	33	34	35	36	37	38	39	46	47	48	49	51	53	54	55	56	57	58
E	F	G	E	E	F	G	E	E	B	B	B	B	B	E	G	G	G	F	E	G	E	F	E	G

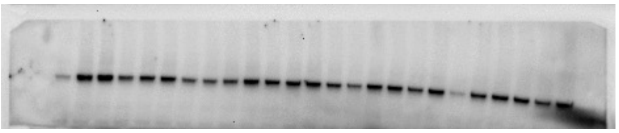

Gel2 MSH3 CTX (1:20K 13E5 Actin)

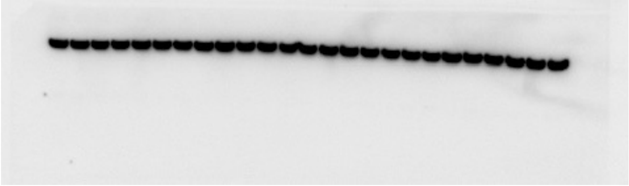

Gel3 MSH3 CTX (1:1K 1F6 MSH3)

26	30	51	54	47	49	33	34	46	25	27	28	29	31	35	36	37	38	39	48	53	55	56	57	58
F	F	F	G	G	G	E	E	E	E	G	E	E	G	B	B	B	B	B	G	E	E	F	E	G

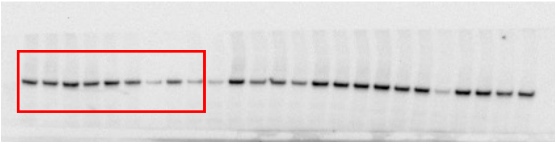

Gel3 MSH3 CTX (1:20K 13E5 Actin)

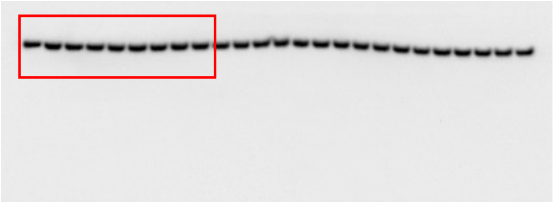

Gel4 MSH3 CTX (1:1K 1F6 MSH3)

29	31	35	36	37	38	39	48	53	55	56	57	58	26	30	51	54	47	49	33	34	46	25	27	28
E	G	B	B	B	B	B	G	E	E	F	E	G	F	F	F	G	G	G	E	E	E	E	G	E

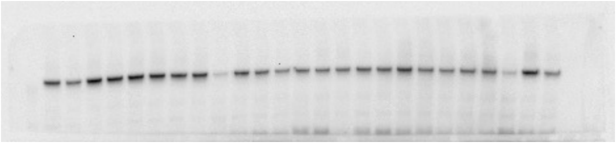

Gel4 MSH3 CTX (1:20K 13E5 Actin)

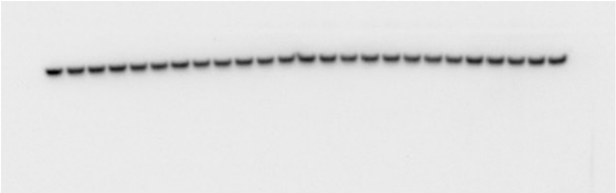

Supplementary Figure 6 Western blot analysis for quantification of cortical MSH3 levels in Hdh$^{Q111/+}$ mice following treatment with vehicle, 3 and 10 nmol MSH3 di-siRNA for 3 months. Four gels were probed with antibodies 1F6 (MSH3) and 13E5 (actin). Groups: B untreated, F vehicle, G 3 nmol and E 10 nmol MSH3 di-siRNA for 3 months (see Figure 2A and B). B and F were combined for cortical MSH3 quantification to compensate for F protein samples lost during processing. Membranes were cut, top probed for MSH3, bottom for actin. Red boxes indicate blots used for Figure 2B (CTX).

Supplementary Table 2 Cortical MSH3 protein levels quantifications

Cortex ID	Group	Gel 1	Gel 2	Gel 3	Gel 4	Average
		% Normalized to (F+B)				
35	B	100.0	97.3	110.4	128.1	109.0
36	B	97.5	86.0	109.9	113.8	101.8
37	B	139.5	104.1	117.7	133.8	123.8
38	B	160.3	97.4	127.8	130.6	129.0
39	B	84.7	63.5	97.8	102.7	87.1
25	E	21.2	22.8	18.1	25.0	21.8
28	E	47.8	79.1	54.6	54.4	59.0
29	E	56.5	104.9	74.7	91.5	81.9
33	E	43.1	55.3	14.9	65.9	44.8
34	E	58.3	58.7	48.3	68.1	58.3
46	E	57.3	44.8	21.5	83.3	51.7
53	E	6.8	16.0	21.0	17.6	15.3
55	E	91.2	94.0	118.2	91.6	98.7
57	E	51.2	47.9	82.9	50.7	58.2
26	F	97.3	137.9	72.5	59.8	91.9
30	F	99.1	109.2	61.1	70.1	84.9
51	F	79.2	103.7	87.0	83.0	88.2
56	F	42.4	100.9	115.9	78.1	84.3
27	G	68.3	170.0	108.5	114.4	115.3
31	G	61.7	58.9	50.3	61.9	58.2
47	G	98.8	90.2	85.8	117.6	98.1
48	G	131.0	107.3	115.9	119.2	118.4
49	G	133.4	68.7	52.7	75.8	82.6
54	G	84.2	71.1	69.6	86.3	77.8
58	G	97.4	50.5	102.3	61.9	78.0

See Supplementary Figure 6 for corresponding Western blots

Supplementary Figure 7

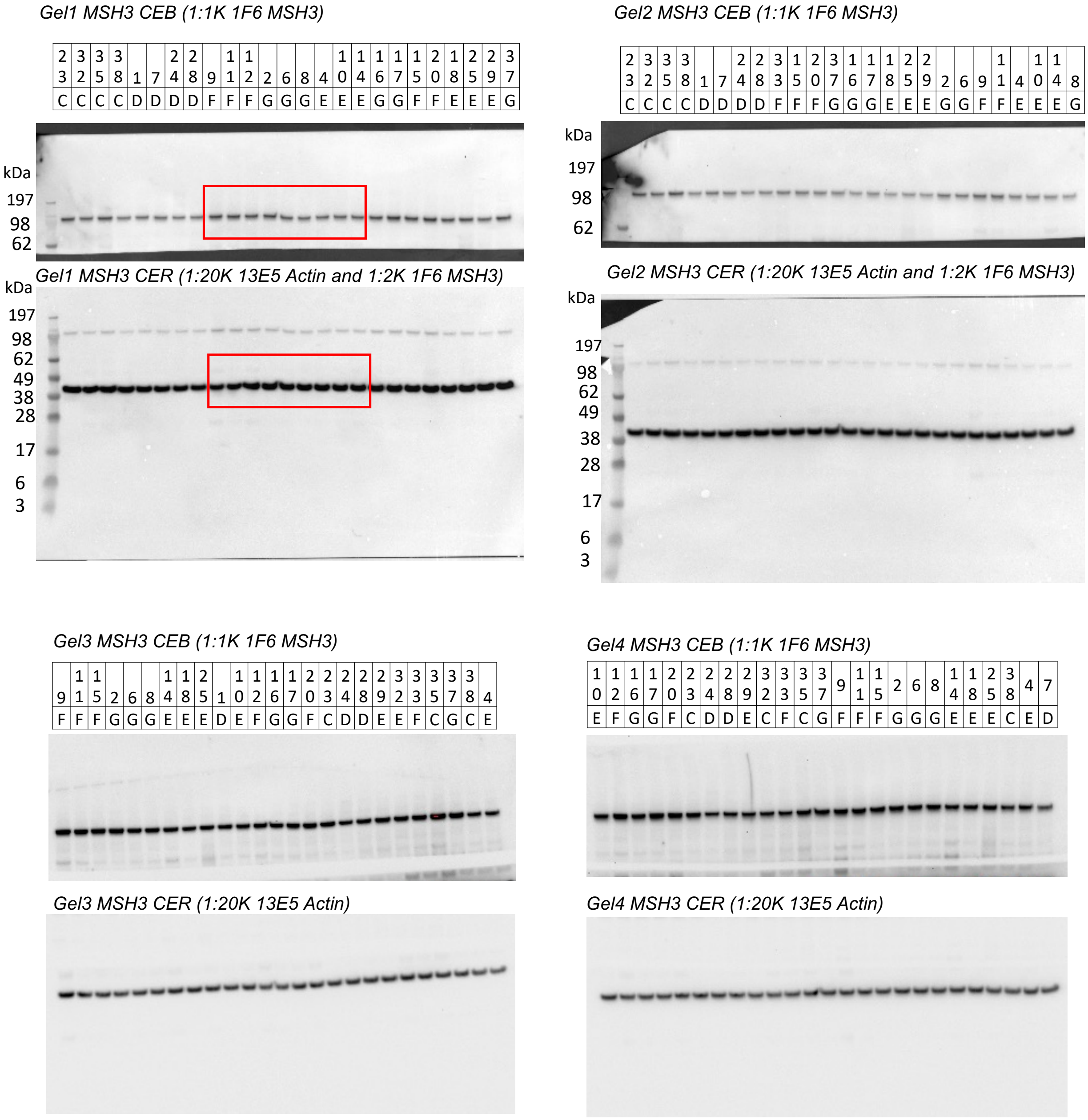

Supplementary Figure 7 Western blot analysis for quantification of cerebellum MSH3 levels in Hdh$^{Q111/+}$ mice following treatment with vehicle, 3 and 10 nmol MSH3 di-siRNA for 2 or 3 months. Four gels were probed with antibodies 1F6 (MSH3) and 13E5 (actin). Groups: C vehicle and D 10 MSH3 di-siRNA nmol for 2 months, F vehicle, G 3 nmol and E 10 nmol MSH3 di-siRNA for 3 months (see Figure 2A and B). Gel 1 and 2 membranes were first probed with both MSH3 (1:2K 1F6) and actin antibodies, then again for MSH3 (1:2K 1F6) after stripping. For gel 3 and 4 membranes were cut, top probed for MSH3, bottom for actin. No protein standard visualized for gel 3 and 4. Red boxes indicate blots used for Figure 2B (CEB).

Supplementary Table 3 Cerebellum MSH3 protein levels quantifications

Cerebellum ID	Group	Gel 1	Gel 2	Gel 3	Gel 4	Average
		% Normalized to F				
23	C	64.0	184.5	91.5	92.3	108.1
32	C	60.4	103.9	78.5	79.4	80.5
35	C	77.7	131.0	136.0	83.5	107.0
38	C	58.8	76.7	76.0	58.0	67.4
1	D	71.6	97.7	76.1		81.8
24	D	67.6	59.9	57.8	57.5	60.7
28	D	76.8	65.6	69.2	75.4	71.7
7	D	80.5	90.2		44.7	71.8
10	E	83.5	98.4	75.3	81.3	84.6
14	E	84.8	95.6	73.3	65.2	79.7
18	E	79.3	61.7	61.2	67.1	67.3
25	E	79.1	74.2	71.9	81.2	76.6
29	E	66.7	72.5	79.1	76.3	73.7
4	E	72.9	95.8	76.8	67.2	78.2
11	F	101.5	120.4	109.0	91.1	105.5
12	F	80.2		100.4	132.5	104.4
15	F	97.1	88.7	102.2	90.1	94.5
20	F	96.7	79.9	103.7	115.9	99.0
33	F		81.9	93.6	75.5	83.7
9	F	124.6	129.1	91.0	95.0	109.9
16	G	99.3	67.4	94.0	104.5	91.3
17	G	110.7	83.6	80.5	121.8	99.1
2	G	79.9	116.5	115.4	77.9	97.4
37	G	78.6	77.2	133.7	88.8	94.6
6	G	81.5	101.5	92.6	75.7	87.8
8	G	73.1	90.9	93.7	79.9	84.4

See Supplementary Figure 7 for corresponding Western blots

Supplementary Figure 8

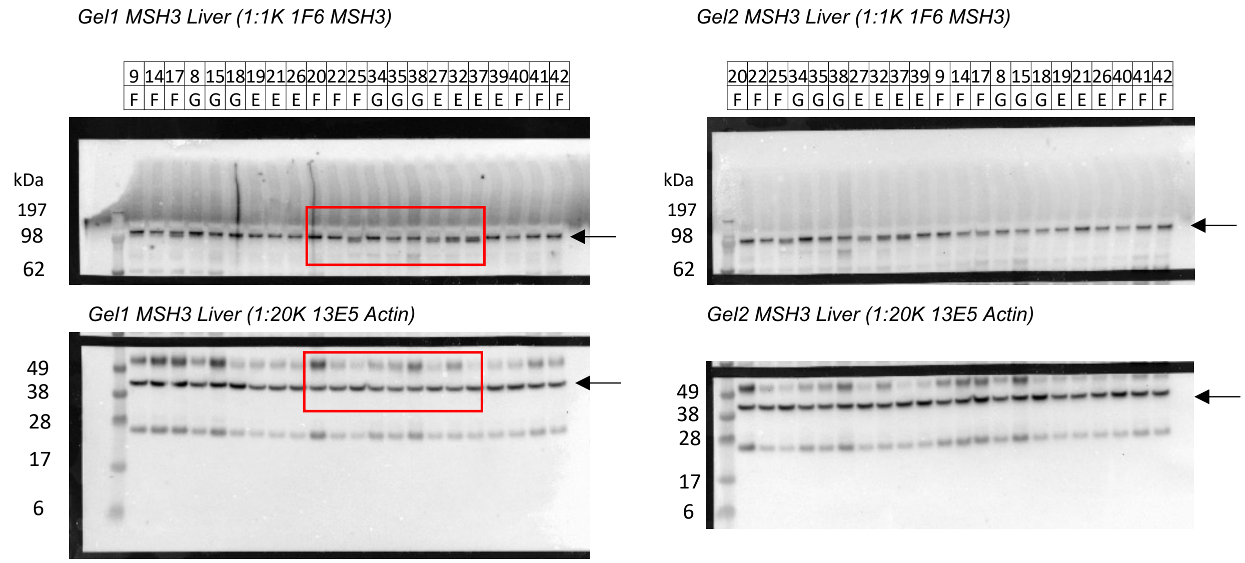

Supplementary Figure 8 Western blot analysis for quantification of liver MSH3 levels in Hdh$^{Q111/+}$ mice following treatment with vehicle, 3 and 10 nmol MSH3 di-siRNA for 3 months. Two gels were probed with antibodies 1F6 (MSH3) and 13E5 (actin). Groups: F vehicle, G 3 nmol and E 10 nmol MSH3 di-siRNA for 3 months (see Figure 2A and B). Membranes were cut, top probed for MSH3, bottom for actin. Red boxes indicate blots used for Figure 2B (LIV)

Supplementary Table 4 Liver MSH3 protein levels quantifications

Liver ID	Group	Gel 1	Gel 2	Average
		% normalized to F		
19	E	104.3	89.8	97.0
21	E	70.9	157.8	114.3
26	E	82.5	87.7	85.1
27	E	73.6	76.6	75.1
32	E	146.7	130.8	138.8
37	E	123.8	120.8	122.3
39	E	95.7	101.8	98.7
9	F	130.6	145.6	138.1
14	F	85.8	76.7	81.3
17	F	70.7	58.9	64.8
20	F	152.2	143.9	148.0
22	F	109.7	98.1	103.9
25	F	76.3	88.7	82.5
40	F	63.9	53.0	58.4
41	F	99.3	102.7	101.0
42	F	111.5	132.3	121.9
8	G	108.9	104.4	106.7
15	G	60.6	63.7	62.2
18	G	78.7	49.0	63.8
34	G	158.1	200.6	179.4
35	G	115.6	150.3	133.0
38	G	117.4	99.5	108.4

See Supplementary Figure 8 for corresponding Western blots

Supplementary Figure 9

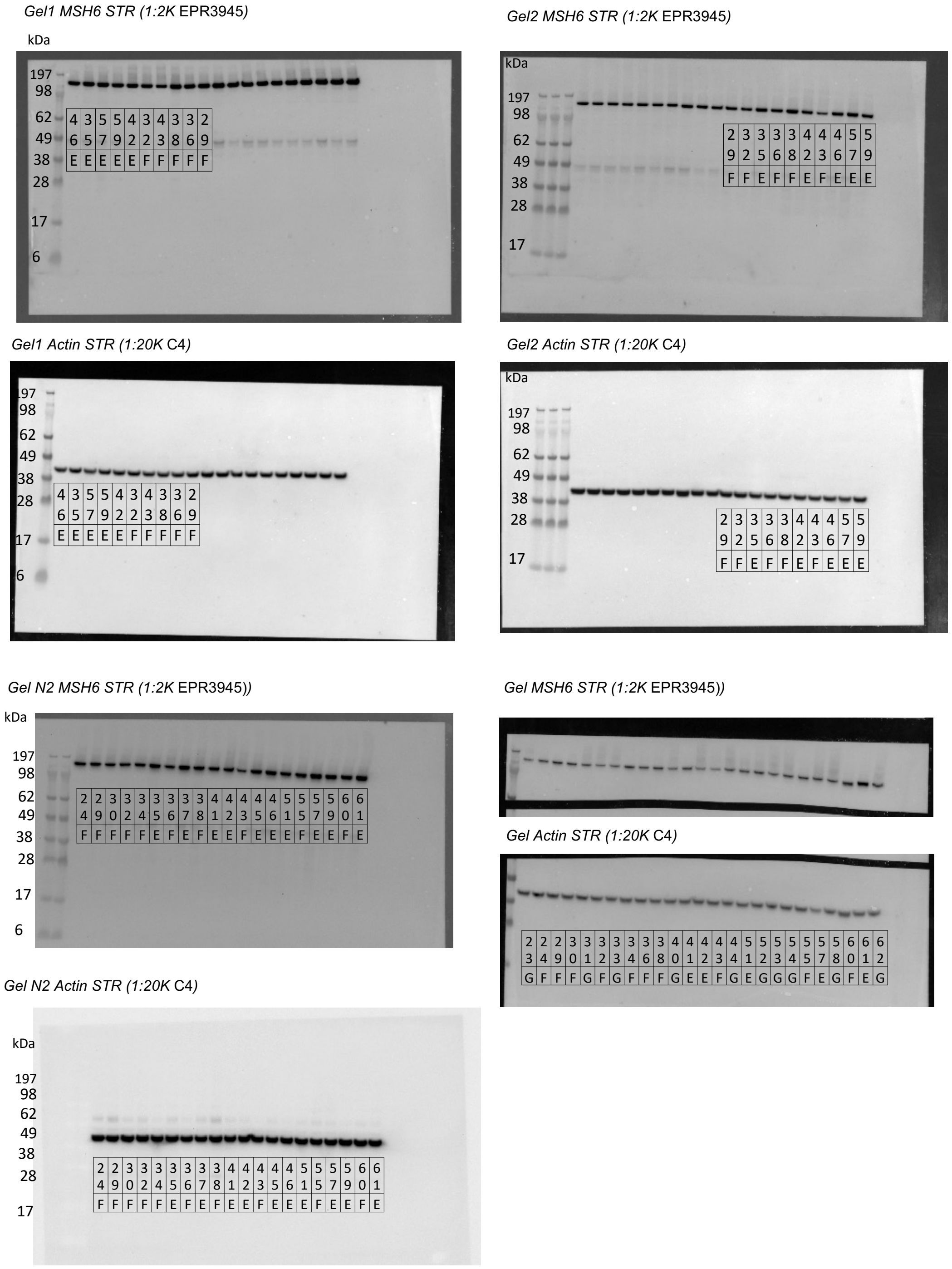

Supplementary Figure 9 Western blot analysis for quantification of striatal MSH6 levels in Hdh$^{Q111/+}$ mice following treatment with vehicle, 3 and 10 nmol MSH3 di-siRNA for 3 months. Gels were probed with antibodies EPR3945 (MSH6) and 13E5 (actin). Groups: F vehicle, G 3 nmol and E 10 nmol MSH3 di-siRNA for 3 months (see Figure 2A and B). Each sample from group G has only been loaded once on a gel.

Supplementary Table 5 Striatum MSH6 protein levels quantifications

Group	Striatum ID	Gel 1	Gel 2	Gel N2	Gel	Average	Average % normalized to F
		% Normalized to F					
G	23				49.8	49.8	49.3
G	31				76.0	76.0	75.2
G	33				70.3	70.3	69.6
G	40				66.1	66.1	65.4
G	44				80.1	80.1	79.3
G	52				109.5	109.5	108.4
G	53				69.9	69.9	69.2
G	54				93.4	93.4	92.4
G	58				99.1	99.1	98.1
G	62				117.8	117.8	116.6
F	24			105.6	77.3	91.4	90.5
F	29	110.7	94	101.0	111.2	104.3	100.5
F	30			74.7	106.2	90.5	89.5
F	32	85.4	72	80.9	54.5	73.2	70.3
F	34			95.5	120.0	107.7	106.7
F	36	108.8	108	90.8	102.9	102.8	98.6
F	38	131.4	174	121.4	132.6	139.9	133.7
F	43	63.7	51	64.8	41.5	55.3	53.2
F	55			137.0	111.5	124.2	123.0
F	60			128.2	142.3	135.3	133.9
E	35	110.5	140	142.5		131.0	124.5
E	37			195.2		195.2	193.2
E	41			138.8	112.0	125.4	124.1
E	42	94.5	106	122.9	48.3	92.9	89.0
E	45			162.6		162.6	160.9
E	46	109.9	137	165.7		137.6	131.1
E	51			128.5	103.4	115.9	114.8
E	57	136.0	168	188.1	165.8	164.6	158.2
E	59	99.4	99	130.5		109.7	104.8
E	61			187.4	250.2	218.8	216.6

See Supplementary Figure 9 for corresponding Western blots

Supplementary Figure 10

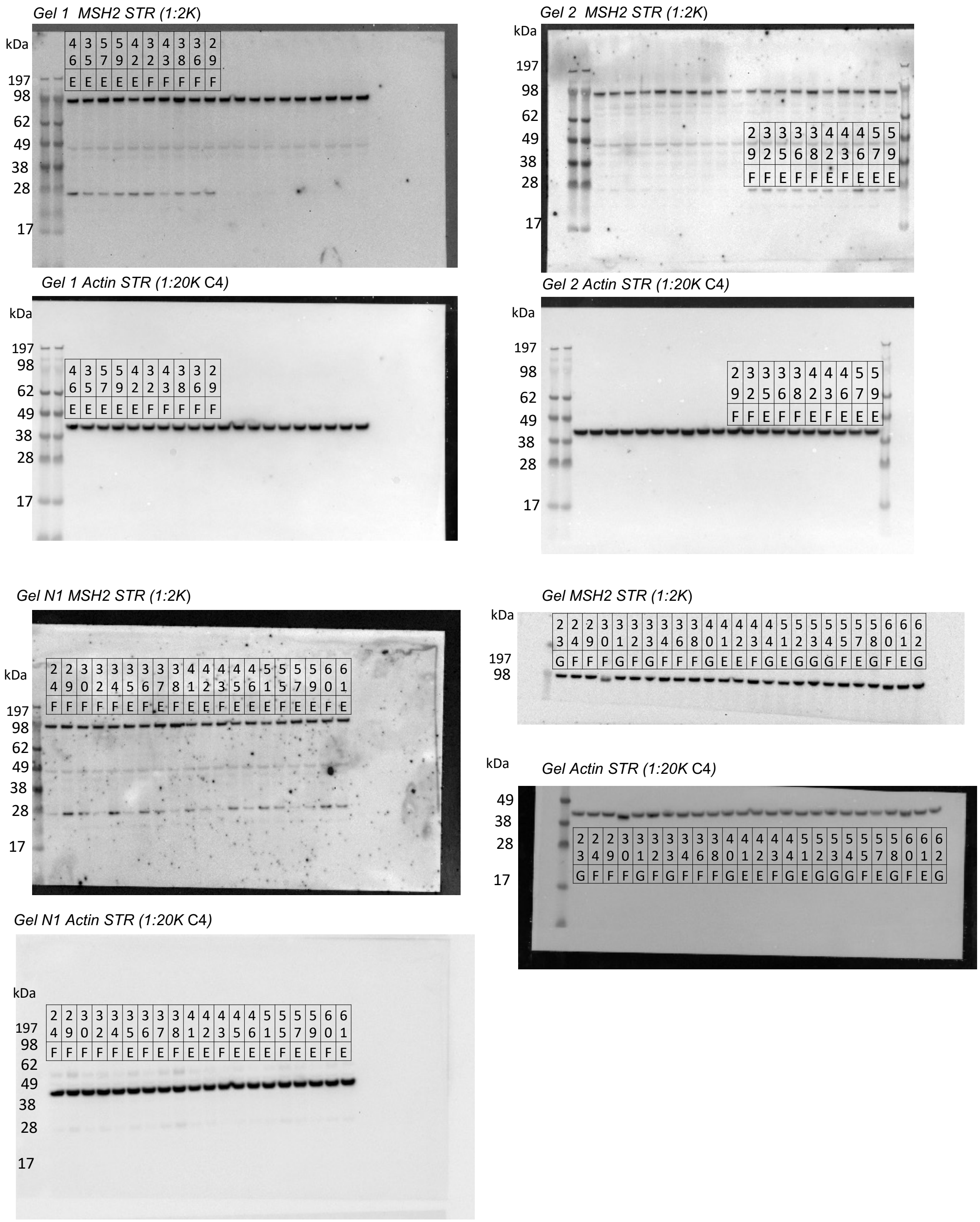

Supplementary Figure 10 Western blot analysis for quantification of striatal MSH6 levels in Hdh$^{Q111/+}$ mice following treatment with vehicle, 3 and 10 nmol MSH3 di-siRNA for 3 months. Gels were probed with antibodies EPR21017-2 (MSH2) and 13E5 (actin). Groups: F vehicle, G 3 nmol and E 10 nmol MSH3 di-siRNA for 3 months (see Figure 2A and B). Each sample from group G has only been loaded once on a gel.

Supplementary Table 6 Striatum MSH2 protein levels quantifications

Group	Striatum ID	Gel 1	Gel 2	Gel N1	Gel	Average	Average % normalized to F
		% Normalized to F					
E	35	79.8	84.9	107.8		90.8	88.4
E	37			121.8		121.8	127.2
E	41			88.6	95.7	92.1	96.5
E	42	61.7	87.2	93.1	79.1	80.3	79.3
E	45			92.2		92.2	96.3
E	46	77.7	112.7	97.6		96.0	92.2
E	51			68.2	88.5	78.4	82.1
E	57	118.0	151.3	119.0	136.5	131.2	129.0
E	59	92.9	123.1	93.8		103.3	98.9
E	61			94.3	141.3	117.8	123.4
F	24			105.4	88.1	96.8	101.3
F	29	95.9	65.2	111.6	96.5	92.3	92.4
F	30			81.0	38.3	59.7	62.4
F	32	87.9	70.2	96.1	68.4	80.6	80.1
F	34			98.0	103.5	100.7	105.5
F	36	87.5	96.3	87.7	95.2	91.7	90.5
F	38	130.3	141.7	138.7	138.7	137.4	135.7
F	43	98.3	126.6	108.4	137.8	117.8	116.3
F	55			89.4	135.4	112.4	117.8
F	60			89.2	98.0	93.6	98.0
G	23				89.3	89.3	93.8
G	31				85.1	85.1	89.3
G	33				72.4	72.4	76.0
G	40				100.2	100.2	105.2
G	44				84.7	84.7	88.9
G	52				118.7	118.7	124.7
G	53				125.3	125.3	131.5
G	54				106.6	106.6	111.9
G	58				120.6	120.6	126.6
G	62				164.7	164.7	173.0

See Supplementary Figure 10 for corresponding Western blots

Supplementary Figure 11

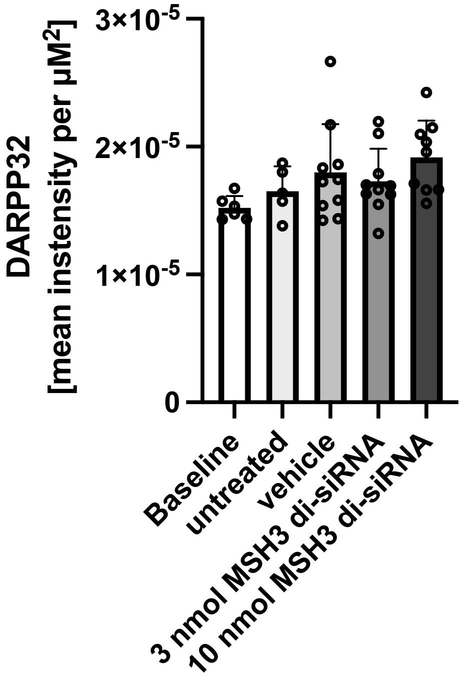

Supplementary Figure 11 Quantification of the DARPP32 mean intensity in the striatum of baseline (3 months of age), vehicle, 3 and 10 nmol MSH3 di-siRNA treated animals and untreated $Hdh^{Q111/+}$ (all 6 months of age). No significant differences were detected. Error bars represent mean ± SD; n=6-10. One-way ANOVA with Tukey's post hoc analysis.

Supplementary Materials & Methods:

FVB/N mice

Female wildtype FVB/N mice were sourced from JAX (strain #001800) at 6-7 weeks old and used at 8 weeks old for pharmacodynamics studies. All procedures on the mice were carried out under approved IACUC protocols at Atalanta Therapeutics (Boston, MA). Mice were maintained in a temperature-controlled space with a 12 hour light/dark cycle. At study end, mice were euthanized via CO2 asphyxiation followed by thoracotomy. Motor cortex, hippocampus, and striatum were collected and immediately frozen on dry ice.

Treatment of FVB/N mice with di-siRNA

Surgical preparation and medication for FVB mice were performed as described for HdhQ111 mice. Mice were treated with 0.5-20 nmol MSH3 di-siRNA, or vehicle (PBS) via bilateral ICV injections. Coordinates for injection were -0.45mm anteroposterior, +/- 1mm mediolateral, and -2.5mm dorsoventral. 5uL of test article was administered to each of the lateral right and left ventricles at a flow rate of 0.5uL/min. Surgical closure was done with wound clips, but recovery was otherwise as described above.

JESS Protein Analysis

For protein analysis from wildtype FVB mice, tissue samples were lysed in 250ul cold Cell Signaling Technology Cell Lysis Buffer supplemented with Halt Protease and Phosphotase Inhibitor Cocktail (ThermoFisher). Samples were homogenized by shaking for two rounds of 2mins at 30Hz using the Qiagen TissueLyser II with 5mm stainless steel beads, followed by a 5min spin at 1000rpm and 30min incubation on ice. Total protein was quantified using the Pierce

BCA Protein Assay Kit, and samples were normalized to 0.125mg/mL with 0.1X Sample Buffer (Jess kit) and prepared according to the ProteinSimple Jess protocol. Samples and antibodies were loaded onto a ProteinSimple 12-230kD separation cartridge, along with all Jess detection module reagents. The primary antibodies used were Millipore Sigma MSH3, clone 1F6 (MABE324), and R&D Actin (MAB8929), mixed together to detect both proteins simultaneously in the same capillary. Bands generated by the Jess were quantified using the Compass for SimpleWestern software, and MSH3 was normalized to Actin for each tissue sample. Samples from the control group were loaded on each gel for normalization purposes.
